# Associations of Intact and C-Terminal FGF23 with Inflammatory Markers in Older Patients Affected by Advanced Chronic Kidney Disease

**DOI:** 10.3390/jcm13133967

**Published:** 2024-07-06

**Authors:** Matteo Abinti, Simone Vettoretti, Lara Caldiroli, Deborah Mattinzoli, Masami Ikehata, Silvia Armelloni, Paolo Molinari, Carlo Maria Alfieri, Giuseppe Castellano, Piergiorgio Messa

**Affiliations:** 1 Department of Nephrology, Dialysis and Renal Transplantation, Fondazione IRCCS Ca’ Granda Ospedale Maggiore Policlinico, 20122 Milan, Italy; matteo.abinti@unimi.it (M.A.); lara.caldiroli@policlinico.mi.it (L.C.); paolo.molinari1@unimi.it (P.M.); carlo.alfieri@unimi.it (C.M.A.); giuseppe.castellano@unimi.it (G.C.); piergiorgio.messa@gmail.com (P.M.); 2 Department of Clinical Sciences and Community Health, University of Milan, 20122 Milan, Italy; 3 Renal Research Laboratory, Fondazione IRCCS Ca’ Granda Ospedale Maggiore Policlinico, 20122 Milan, Italy; deborah.mattinzoli@policlinico.mi.it (D.M.); masami.ikehata@policlinico.mi.it (M.I.); silvia.armelloni@policlinico.mi.it (S.A.)

**Keywords:** chronic kidney disease, fibroblast growth factor 23, inflammation, cytokines

## Abstract

**Background**: In patients with chronic kidney disease (CKD), Fibroblast Growth Factor 23 (FGF23) is markedly increased and has been proposed to interact with systemic inflammation. **Methods**: In this cross-sectional study, we evaluated the correlations of intact FGF23, c-terminal FGF23, and the FGF23 ratio (c-terminal to intact) with some inflammatory cytokines in 111 elderly patients with advanced CKD not yet in dialysis. **Results**: Estimated glomerular filtration rate (eGFR) was inversely correlated with intact FGF23 and c-terminal FGF23, as well as with interleukin 6 (IL-6), tumor necrosis factor alpha (TNFα), and monocyte chemoattractant protein-1 (MCP-1). Intact FGF23 levels were directly correlated with IL-6 (r = 0.403; *p* < 0.001) and TNFα (r = 0.401; *p* < 0.001) while c-terminal FGF23 was directly correlated with MCP-1 (r = 0.264; *p* = 0.005). The FGF23 ratio was, instead, inversely correlated with IL-6 (r = −0.326; *p* < 0.001). Multivariate analysis revealed that intact FGF23 was directly associated with TNFα [B = 0.012 (95% CI 0.006, 0.019); *p* = 0.003] and c-terminal FGF23 was directly associated with MCP-1 [B = 0.001 (95% CI 0.000, 0.002); *p* = 0.038], while the FGF23 ratio was inversely correlated with IL-6 [B = −0.028 (95% CI −0.047, −0.010); *p* = 0.002]. **Conclusions**: Our data demonstrate that, in CKD patients, intact FGF23 and the metabolites deriving from its proteolytic cleavage are differently associated with some inflammatory pathways. In particular, intact FGF23 is mainly associated with IL-6 and TNFα, c-terminal FGF23 with MCP-1, and the FGF23 ratio with IL6.

## 1. Introduction

Chronic kidney disease (CKD) affects more than 10% of the general population worldwide (approximately 850 million individuals), and its prevalence is constantly increasing [1]. It requires expensive renal replacement therapies when end-stage kidney disease (ESKD) occurs, and it is burdened by increased cardiovascular (CV) and mortality risks [2]. Beyond the traditional causal factors for cardiovascular disease (CVD) and mortality in CKD [3], a position of interest has long been occupied by mineral and metabolic alterations [4]. Furthermore, CKD patients are affected by persistent low-grade inflammation that is secondary to the dysregulation of the balance between pro- and anti-inflammatory effectors [5,6,7]. The persistence of systemic inflammation may result in a progressive decline in eGFR [5] as well as in an increase in CV morbidity and mortality [8].

Fibroblast Growth Factor 23 (FGF23), mainly produced and secreted from bone cells, has been formerly described as a phosphate-regulating hormone. This function is carried out by reducing phosphate renal reabsorption and by reducing its intestinal absorption and bone resorption through the inhibition of both calcitriol and PTH synthesis and release. High FGF23 levels not only contribute to the development of mineral and bone disease (MBD) but in some studies they have been proposed to play a causative role in the increased CV risk of CKD patients [9]. In fact, FGF23 levels rise since the very early stages of CKD, and their remarkable increase along CKD progression is secondary to the need to maintain a neutral phosphate balance [10]. However, FGF23 increase may also depend on an altered cleavage from the intact form to its fragments, as well as on the reduction in its renal clearance [11].

Many experimental and observational studies suggest that FGF23 and inflammation have a reciprocal bidirectional interrelationship. In fact, FGF23 can induce or at least worsen the inflammatory status [12,13,14,15], whereas inflammation can interfere with the secretion and catabolism of FGF23 through multiple pathways [16,17,18]. Several proinflammatory cytokines, including Tumor Necrosis Factor α (TNFα) and Interleukin 6 (IL-6), appear to play a role in FGF23 increase [19], although it is still uncertain whether they are mostly related to changes in the synthesis and/or cleavage of FGF23 [15,20]. Recent studies have also suggested that the increased production of Monocyte Chemoattractant Protein 1 (MCP-1) may be associated with FGF23 metabolism and contribute to CKD progression by promoting monocyte recruitment and their conversion to macrophages in the kidneys [21,22]. Moreover, other factors indirectly related to inflammation, such as functional iron deficiency and anemia [23], have been purported to affect FGF23 transcription and cleavage from the full intact molecule (iFGF23) into its fragments, such as the C-terminal end of the FGF23 molecule (cFGF23), that is involved in other biological actions and active in different biological pathways [16,23]. Usually, in non-CKD patients, these complex interactions cause a rise in serum c-terminal FGF23 levels with little to no impact on intact FGF23 [24]. Nevertheless, in CKD patients, intact FGF23 cleavage is impaired by unknown mechanisms; therefore, as the estimated glomerular filtration rate (eGFR) declines, circulating intact FGF23 increases faster than c-terminal FGF23 [25].

To date, the literature agrees that inflammation, anemia, and iron deficiency contribute to higher FGF23 levels, but it is still unclear if and which specific biomarker(s) is(are) associated with the synthesis [25,26] and/or cleavage of this hormone [27,28,29].

Hence, in order to provide a more complete interpretation of the causes that lead to higher FGF23 levels in CKD patients, it has been proposed to assess both intact and c-terminal FGF23 levels contemporarily, since the FGF23 ratio may represent the balance between FGF23 transcription and proteolytic cleavage in such a clinical setting [25,29].

In this study, we investigated whether there was any association between the parameters intercepting FGF23 metabolism (intact FGF23, c-terminal FGF23, and the FGF23 ratio) and some specific biomarkers of systemic inflammation and iron metabolism in a group of elderly CKD patients not on dialysis.

## 2. Materials and Methods

### 2.1. Patients and Design of the Study

Among a total of 432 prevalent CKD patients attending our outpatient CKD clinic between 9/2016 and 3/2018, we selected 111 individuals. The selection criteria were age ≥ 65 years, CKD stages 3a to 5 not yet on dialysis, and a relatively stable eGFR (estimated according to the CKD-EPI formula [30]) with less than 2 mL/min/1.73/m^2^ of variation over the previous 6 months.

To limit the possible confounding factors, we excluded patients unable to cooperate as well as those with cancer, cirrhosis, severe heart failure (NYHA class II–IV), nephrotic syndrome, thyroid diseases, bowel inflammatory diseases, and primary or tertiary hyperparathyroidism (overall 147 patients). We also excluded patients treated with erythropoiesis-stimulating agents, oral or intravenous iron compounds, immunosuppressive drugs, or who had been hospitalized in the previous 3 months (overall 118 patients). Finally, among the remaining 167 patients, 56 were excluded because of incomplete data collection.

Clinical data and urinary and biochemical parameters were collected simultaneously, with single measurements for every patient, at the index visit. Biochemical and urinary sampling was performed in the morning after an overnight fast of at least 12 h. Basic biochemical analyses were performed in the central laboratory of our Institution as per the common indication of clinical practice in patients with CKD. The study was conducted according to the ICP Good Clinical Practices Guidelines and was approved by the Ethics Committee of our Institution (Milano 2-approval N. 347/2010). All patients provided informed consent before participation.

### 2.2. Specific Biomarkers Quantification

All samples of interest for this study were drawn at the time of the index visit and then frozen and preserved at −80 °C.

Plasmatic intact FGF23 and c-terminal FGF23 levels were measured using a second-generation two-site enzyme-linked immunosorbent assay ELISA Kit (Immutopics Quidel Co., San Diego, CA, USA), as performed in previous studies [31,32]. The minimal detectable concentrations were 1.5 pg/mL and 1.5 RU/mL, respectively. The coefficients of variation were as follows: (i) intact FGF23: intra-assay 4.1% and 2% at 43 and 426 pg/mL and inter-assay 9.1% and 3.5% at 46 and 441 pg/mL; (ii) c-terminal FGF23: intra-assay 2.4% and 1.42% at 33.7 and 302 RU/mL and inter-assay 1.4% at 33.6 and 293 RU/mL, respectively. The absorbance in each well was read at a dual wavelength of 450/630 nm. The FGF23 ratio was calculated as c-terminal to intact FGF23.

Cytokine concentrations were determined using enzyme-linked immunosorbent assay (ELISA) kits following the manufacturer’s instructions. The following kits were used: Human TNFa ELISA Kit (Thermo Fisher Scientific, Monza, Italy) with a declared kit sensitivity of <2 pg/mL. For IL-6 dosage, 3 different ELISA kits, with standard curve ranges of decreasing values, were used and compared: Human IL-6 ELISA Kit EH2IL6 (Thermo Fisher Scientific, Monza, Italy), Human IL-6 Platinum ELISA BMS213/2 (A ymetrix, Thermo Fisher Scientific, Monza, Italy), and Quantikine HS ELISA Human IL-6 Immunoassay HS600B (R&D Systems, Space, Milano, Italy), with a sensitivity of <1 pg/m, 0.92 pg/mL and 0.110 pg/mL, respectively. For IL-6 quantification, Quantikine HS ELISA, Human IL-6 Immunoassay HS600B, and Human IL-6 ELISA Kit EH2IL6 results were compared using a simple regression test, and both results were indifferently used after determining the significant correlation.

Serum MCP-1 levels, considered as a marker of inflammation, were evaluated using a commercially available ELISA Kit (R&D Systems, Inc., Minneapolis, MN, USA) [33,34]. The minimal detectable concentration was 1.7 pg/mL. The coefficients of variation for the intra-assay were 7.8% and 4.7% at 76.7 and 364 pg/mL, respectively, and for the inter-assay, 6.7% and 5.8% at 74.2 and 352 pg/mL, respectively. The absorbance in each well was read at a dual wavelength of 450/570 nm. For both ELISA, the replicate background measurements were subtracted from all 450 nm measurements [35].

In each test, the curve included zero as the last standard point. Quantikine Immunoassay Control Groups 1–4 or 10 (R&D Systems, Space, Milano, Italy), as appropriate, were used to assess assay acceptability. Absorbance readings were measured at 450 nm using a spectrophotometer (Xenius Safas, Monaco). All cytokine values were evaluated in duplicate.

### 2.3. Normal Range of Laboratory Parameters

We used reference values from the central laboratory of our Institution to examine routine parameters related to eGFR, osteo-mineral, and iron metabolism.

For inflammatory cytokines (except for C-reactive protein (CRP)), because there were no univocal normal levels, we considered the values of healthy controls of the same age as those reported in a previous study from our group [35].

For intact FGF23, we set the normal range considering the plasmatic levels from 15 healthy subjects matched for age (preserved eGFR, range 81 ± 7 mL/min, age 75 ± 5 years). For c-terminal FGF23, the normal range refers to a recent study by Smith et al. [25].

The normality ranges considered for all variables are listed in Table 1.

### 2.4. Statistical Analysis

Continuous variables were expressed as mean ± standard deviation (SD) in parametric distributions or as median ± interquartile range (IQR) in non-parametric data. Categorical variables are reported as percentages. Some non-parametric variables (sCr, eGFR, proteinuria, PTH, intact FGF23, c-terminal FGF23, ferritin, CRP, IL-6, TNFα, and MCP-1) were logarithmically transformed with base ten.

Bivariate analyses (with two-tailed Spearman bivariate analysis) and multivariate analyses (with multiple linear regression models) were used to analyze the data of our population and predict FGF23 levels. Negative correlations are expressed with the prefix “-”. In multivariate analyses, we ran multiple analyses that considered the parameters that showed statistical significance for intact FGF23, c-terminal FGF23, or the FGF23 ratio at previous univariate analyses. Three different models were built, considering intact FGF23, c-terminal FGF23, and the FGF23 ratio separately as dependent variables. Variance inflation factors (VIFs) were considered in the construction of the models. Only the variables that remained relevant were reported in our results, in hierarchical order according to their statistical significance.

A *p* < 0.05 was considered statistically significant for all analyses.

All analyses were performed using SPSS 21 (IBM, Armonk, NY, USA).

## 3. Results

### 3.1. General and Biochemical Characteristics of the Study Group

Patients’ characteristics are summarized in Table 1. The mean age was 77 ± 12 years, 71% were males, and the mean BMI was 27.8 ± 4.8 Kg/m^2^. The median eGFR was 23 (16–31) mL/min, whereas the median proteinuria was 0.5 (0.2–1.3) g/24 h. Considering comorbidities, 88% of patients were hypertensives, and 55% were diabetics.

Hypertensive nephropathy was the most frequent cause of CKD, involving 37% of our population, followed by diabetic nephropathy (28%) and glomerular disease (11%). In about 20% of patients, we did not identify an etiologic cause of CKD.

### 3.2. Correlations of FGF23 Isoforms with General and Laboratory Parameters

The correlations of FGF23 isoforms with the main general characteristics of the population are reported in Table 2.

Overall, c-terminal FGF23 levels and the FGF23 ratio were directly correlated with patients’ age. Both intact and c-terminal FGF23, but not the FGF23 ratio, were inversely correlated to eGFR. Supplementary Figure S1 shows the correlation graphs between eGFR and FGF23 isoforms.

Moreover, intact and c-terminal FGF23 directly correlated to proteinuria, PTH, and s-P levels. Intact FGF23 was correlated with s-Ca and 25-OH-Vit-D by an inverse and direct relationship, respectively. C-terminal FGF23 was directly correlated with u-P, whereas the FGF23 ratio was inversely associated with 25-OH-Vit-D.

Considering the significant correlations with inflammatory cytokines, intact FGF23 was positively correlated with IL-6 (r = 0.403; *p* < 0.001) and TNFα (r = 0.401; *p* < 0.001); c-terminal FGF23 was positively correlated with MCP-1 (r = 0.264; *p* = 0.005), whereas the FGF23 ratio was negatively correlated with IL-6 (r = −0.326; *p* < 0.001). Correlations between FGF23 and inflammatory biomarkers are also reported as scatter plots in Supplementary Figure S2.

Regarding the parameters related to erythropoiesis and iron metabolism, intact FGF23 was significantly and negatively correlated with Hb, serum iron, and TSAT, whereas c-terminal FGF23 and the FGF23 ratio were significantly and inversely correlated with Hb and ferritin, respectively. No significant correlation was found between FGF23 and the identified CKD etiologies (*p* = 0.917 for intact FGF23, and *p* = 0.585 for c-terminal FGF23). Furthermore, we did not find significant correlations between FGF23 and diabetes (*p* = 0.466 for intact FGF23, and *p* = 0.355 for c-terminal FGF23).

### 3.3. Correlations of Inflammatory Cytokines with General and Laboratory Parameters

To better explore the role of inflammation in our patients, we then looked for the correlations between IL-6, TNFα, and MCP-1 and the main parameters considered. We quote here only the most significant ones; but complete information is listed in Table 3.

We found a significant inverse correlation of IL-6, TNFα, and MCP-1 with eGFR. These relationships are also graphically represented in Supplementary Figure S3.

Moreover, IL-6 showed a significant direct correlation with s-P, PTH, and uP, while it had an inverse correlation with Serum Iron and TSAT. TNFα showed a significant direct correlation with s-P and 25-OH Vitamin D, while it had an inverse correlation with Serum Albumin and Hemoglobin. MCP-1 showed a direct correlation with BMI and PTH. We did not find significant correlations of inflammatory cytokines with diabetes (*p* = 0.505 for IL6, *p* = 0.513 for TNFα, and *p* = 0.546 for MCP-1), or with CKD etiologies.

Lastly, correlating every inflammatory cytokine with the others, we found a direct statistically significant correlation between IL6 and TNFα (r = 0.254, *p* = 0.007). On the contrary, we interestingly found that MCP-1 did not correlate with any other inflammatory cytokines.

### 3.4. Multivariate Analyses

Lastly, we built multivariate linear regression models by performing separate analyses in which intact FGF23, c-terminal FGF23, and the FGF23 ratio were considered as dependent variables. These isoforms were correlated with inflammatory cytokines and elements of bone-mineral, erythropoiesis, and iron metabolism.

In the construction of each model, we initially considered the variables that showed a strong statistical significance in the univariate analyses, (i.e., eGFR, age, PTH, and s-P), also considering their proved causal relation to FGF23 and inflammation, as is well described in the literature. By multiple attempts (not reported here), we progressively tried to add to the models other significant variables (according to the univariate results from our population).

Table 4 reports our final models, that list in hierarchical order only the variables that maintained statistical significance in multivariate regression. A significant proportion of the variance of intact FGF23 was explained by a model including eGFR, s-P, PTH, and TNFα; [R2 = 0.509, F(4/111) = 27.179, *p* < 0.001]. For c-terminal FGF23, the model included eGFR, s-P, MCP-1, and age; [R2 = 0.363, F(4/111) = 14.969, *p* < 0.001]. Finally, the regression model for the FGF23 ratio included 25-OH vitamin D, age, and IL-6; [R2 = 0.233, F(3/111) = 10.864, *p* < 0.001].

These multivariate analyses showed that TNFα maintained its positive correlation with intact FGF23 [B = 0.012 (95%CI 0.006, 0.019); *p* = 0.003], while IL-6 maintained its negative correlation with the FGF23 ratio [B = −0.028 (95%CI −0.047, −0.010); *p* = 0.002]. On the other hand, MCP-1 confirmed its positive correlation with c-terminal FGF23 [B = 0.001 (95%CI 0.000, 0.002 *p* = 0.038].

eGFR and s-P maintained their significance for both intact and c-terminal FGF23. Age appeared to be strongly relevant for c-terminal FGF23 and the FGF23 ratio. Interestingly, intact FGF23 better correlated with PTH, while the FGF23 ratio with 25-OH Vitamin D. Regarding the variables related to erythropoiesis and iron metabolism, apparently none of the statistically significant correlations initially found in the univariate analyses maintained their significance in our regression models.

## 4. Discussion

Our data confirm that in CKD patients, there is a significant increase in both intact and c-terminal FGF23; however, no significant change was observed in their ratio. Furthermore, CKD has been reported to be associated with low-grade inflammation due to many factors, including an altered immune response [5] and malnutrition [36,37,38], that lead to an increased production and accumulation of inflammatory cytokines. Our data, in this sense, confirm that the progressive worsening of eGFR is associated with increased levels of several proinflammatory cytokines (IL-6, TNFα, and MCP-1).

More recently, FGF23 has also been demonstrated to be bidirectionally associated with systemic inflammation, since many compounds with inflammatory properties can stimulate its production and, on the other hand, high levels of FGF23 can promote the synthesis of some inflammatory cytokines [15]. For this reason, we explored whether the increase in FGF23 and cytokines was interrelated. Univariate analysis revealed that intact FGF23 levels were significantly and directly related to TNFα and IL-6, but not to MCP-1, whereas c-terminal FGF23 was positively related to MCP-1 levels. These results suggest that some peculiarities characterize the patterns of FGF23 metabolic control by different proinflammatory cytokines, with some mainly increasing the intact form of this hormone, whereas others prevailingly stimulating its proteolytic cleavage. These aspects could not be trivial from a clinical point of view, given the possible interconnections of inflammation and FGF23 with cardiovascular and skeletal systems, at least in the clinical setting of CKD [15,39]. Only future experimental and clinical studies will consent to better dissect these complicated and intriguing pathways.

Recent evidence has proposed that FGF23 could be involved in the association with indirect markers of inflammation, such as functional iron deficiency (particularly TSAT), anemia with an increased need of EPO, and death [40]. In our population, intact and c-terminal FGF23 showed a strong inverse correlation with Hb levels. Moreover, iFGF23 revealed a positive correlation with serum iron and TSAT. Nevertheless, none of these correlations between FGF23 isoforms and the parameters related to erythropoiesis and/or iron metabolism remained significant in multivariate analyses. These results, in contrast with those of other reports [41,42,43,44], may depend on methodological and group-related differences.

To the best of our knowledge, our study is the first one to correlate different FGF23 isoforms with inflammatory cytokines and parameters of iron metabolism and erythropoiesis in a global vision. In this regard, our results suggest that the relationship between erythropoiesis and iron metabolism on one hand and the metabolism of FGF23 on the other could both depend on inflammatory status, but without being directly interrelated to each other. Again, it seems that inflammatory cytokines independently correlate with FGF23 isoforms, suggesting that they possibly interact through different pathological pathways.

Our study has several limitations. First, its observational and cross-sectional design does not allow to prove or disprove any cause/effect relationship between the evaluated variables. Second, the study population has some specific age- and ethnic-related specificity (all patients were Caucasian) that makes it difficult to generalize our results to different populations. Third, our results, although significant, were based on single measurements that may at least depend on contingent conditions and that are therefore weaker than those obtained by repeated measurements. Finally, in some cases, statistical significance was associated with a small degree of correlation (r < 0.5), this might depend on the relative small number of patients as well as on the presence of other unrecognized and not evaluated conditions that could have influenced these correlations.

However, this study also has some equally relevant strengths that are due to the strict selection criteria of the evaluated population that limits the possible sources of bias and the accuracy and completeness in the assessment of the specific parameters that may influence FGF23 metabolism. Therefore, this should improve the overall quality of our study and strengthen the relevance of the correlations that we found.

## 5. Conclusions

Our results show that in older patients affected by CKD, intact and C-terminal FGF23 isoforms are differently associated with inflammation and with relevant parameters of iron metabolism and erythropoiesis.

In perspective, our results could help to generate new hypotheses on FGF23 metabolism based on its association with systemic inflammation and possibly with clinical outcomes.

## Figures and Tables

**Table 1:** General and Biochemical Characteristics of the Population.

General characteristics		n. v.
Age (years)	77 + 12	n.a.
BMI (Kg/m^2^)	27.8 + 4.8	19–25
Sex (M/F), %	71/29	n.a.
Hypertension (Yes/No), %	88/12	n.a.
Diabetes mellitus (Yes/No), %	55/45	n.a.
sCr (mg/dL)	2.4 (1.9–3.1)	0.72–1.18
eGFR (mL/min/1.73 m^2^)	23 (16–31)	>90
Proteinuria (g/24 h)	0.5 (0.2–1.3)	<0.14
Serum albumin (g/dL)	4.1 + 0.4	3.4–4.8
Serum prealbumin (mg/dL)	29 + 6	20–40
Bone-mineral metabolism		
s-Ca (mg/dL)	9.2 + 0.5	8.4–10.2
Ca++ (mg/dL)	4.7 + 0.5	4.8–5.6
s-P (mg/dL)	3.6 + 0.6	2.7–4.5
PTH (pg/mL)	61 (39–92)	6.5–36.8
25-OH Vitamin D (ng/mL)	26 (17–38)	>30
iFGF23, (pg/mL)	117 (69–202)	25–45
cFGF23, (RU/mL)	133 (96–229)	20–90
u-Ca (mg/24 h)	118 + 73	100–300
u-P (mg/24 h)	506 + 206	400–1300
Erythropoiesis and iron metabolism		
Hb, g/dL	12.5 + 1.5	13.5–17.5
Serum iron (mcg/dL)	71 (56–93)	59–158
Ferritin (mcg/L)	118 (69–199)	30–400
Transferrin/mg/dL)	231 + 40	200–360
TSAT (%)	23.5 + 8.5	15–50
Inflammatory markers		
CRP (mg/dL)	0.2 (0.1–0.4)	<0.5
IL-6 (pg/mL)	3.1 (1.6–5.4)	0–17.3
TNFα (pg/mL)	13.7 (9.3–18.5)	1.5–20.5
MCP-1 (pg/mL)	339 (270–515)	199–486

Notes: BMI = Body Mass Index; sCr = serum creatinine; eGFR = estimated Glomerular Filtration Rate; s-Ca = serum calcium; Ca++ = serum ionized calcium; s-P = serum phosphorus; PTH = Parathormone; iFGF23 = intact FGF23; cFGF23 = c-terminal FGF23; u-Ca = 24 h urinary calcium excretion; u-P = 24 h urinary phosphorus excretion; Hb = Hemoglobin; TSAT = Transferrin Saturation; CRP = C-Reactive Protein; IL-6 = Interleukin 6; TNFα = Tumor Necrosis Factor a; MCP-1 = Monocyte Chemoattractant Protein 1.

**Table 2:** Associations of FGF23 isoforms with general and laboratory parameters.

	iFGF23 (pg/mL)		cFGF23 (RU/mL)		FGF23 Ratio (RU/pg)	
	r	*p*	r	*p*	r	*p*
General characteristics						
Age (years)	0.016	0.866	0.235	**0.013**	0.239	**0.011**
BMI (kg/m^2^)	−0.117	0.223	−0.060	0.531	0.054	0.571
eGFR (mL/min/m^2^)	−0.605	**<0.001**	−0.483	**<0.001**	0.127	0.113
Proteinuria (g/24 h)	0.242	**0.013**	0.225	**0.022**	−0.086	0.388
Serum albumin (g/dL)	−0.081	0.397	−0.129	0.177	−0.086	0.368
Serum prealbumin (mg/dL)	0.077	0.424	0.014	0.886	−0.132	0.167
Bone-mineral metabolism						
s-Ca (mg/dL)	−0.178	**0.049**	−0.104	0.289	0.019	0.843
s-P (mg/dL)	0.448	**<0.001**	0.343	**<0.001**	−0−127	0.187
PTH (pg/mL)	0.451	**<0.001**	0.369	**<0.001**	−0.132	0.168
25-OH Vitamin D (ng/mL)	0.197	**0.038**	−0.025	0.797	−0.267	**0.005**
uCa (mg/24 h)	−0.058	0.556	0.094	0.343	0.108	0.277
uP (mg/24 h)	−0.113	0.254	−0.268	**0.006**	−0.160	0.107
Erythropoiesis and iron metabolism						
Hb (g/dL)	−0.288	**0.002**	−0.287	**0.002**	−0.018	0.849
Serum iron (mcg/dL)	−0.218	**0.023**	−0.092	0.341	0.125	0.194
Ferritin (μg/dL)	0.074	0.441	−0.074	0.444	−0.169	**0.048**
Transferrin (mg/dL)	−0.132	0.170	0.016	0.867	0.125	0.194
TSAT (%)	−0.161	**0.038**	−0.098	0.308	0.071	0.464
Inflammatory markers						
CRP (mg/dL)	−0.033	0.732	−0.056	0.557	0.051	0.598
IL-6 (pg/mL)	0.403	**<0.001**	0.176	0.064	−0.326	**<0.001**
TNFα (pg/mL)	0.401	**<0.001**	0.110	0.251	−0.183	0.045
MCP-1 (pg/mL)	0.150	0.152	0.264	**0.005**	0.178	0.061

Notes: BMI = Body Mass Index; sCr = serum creatinine; eGFR = estimated Glomerular Filtration Rate; s-Ca = serum calcium; Ca++ = serum ionized calcium; s-P = serum phosphorus; PTH = Parathormone; iFGF23 = intact FGF23; cFGF23 = c-terminal FGF23; uCa = 24 h urinary calcium excretion; uP = 24 H urinary phosphorus excretion; Hb = Hemoglobin; TSAT = Transferrin Saturation; CRP = C-Reactive Protein; IL6 = Interleukin 6; TNFα = Tumor Necrosis Factor a; MCP-1 = Monocyte Chemoattractant Protein 1. Bold highlights statistical significances (*p* < 0.05).

**Table 3:** Associations of inflammatory cytokines with general and laboratory parameters.

	IL-6 (pg/mL)		TNFα (pg/mL)		MCP-1 (pg/mL)	
	r	*p*	r	*p*	r	*p*
General characteristics						
Age (years)	0.116	0.227	0.057	0.551	0.154	0.106
BMI (kg/m^2^)	0.055	0.569	−0.056	0.559	0.291	**0.002**
eGFR (mL/min/m^2^)	−0.288	**0.002**	−0.286	**0.002**	−0.238	**0.012**
Proteinuria (g/24 h)	0.088	0.376	0.174	0.077	0.046	0.642
Serum albumin (g/dL)	−0.162	0.089	−0.196	**0.039**	−0.097	0.310
Serum prealbumin (mg/dL)	−0.079	0.412	−0.089	0.354	−0.142	0.137
Bone-mineral metabolism						
s-Ca (mg/dL)	−0.039	0.687	0.059	0.540	0.026	0.788
s-P (mg/dL)	0.200	**0.036**	0.299	**0.002**	0.052	0.591
PTH (pg/mL)	0.323	**<0.001**	0.089	0.350	0.215	**0.024**
25-OH Vitamin D (ng/mL)	0.034	0.720	0.201	**0.035**	−0.020	0.835
uCa (mg/24 h)	−0.121	0.222	0.097	0.326	0.040	0.690
uP (mg/24 h)	−0.210	**0.033**	−0.117	0.241	−0.151	0.129
Erythropoiesis and iron metabolism						
Hb (g/dL)	−0.105	0.273	−0.198	**0.037**	−0.146	0.127
Serum iron (mcg/dL)	−0.326	**<0.001**	−0.146	0.130	−0.113	0.243
Ferritin (μg/dL)	0.100	0.297	0.054	0.573	−0.114	0.235
Transferrin (mg/dL)	−0.137	0.152	−0.073	0.451	−0.146	0.127
TSAT (%)	−0.231	**0.016**	−0.014	0.239	−0.017	0.858

Notes: BMI = Body Mass Index; sCr = serum creatinine; eGFR = estimated Glomerular Filtration Rate; s-Ca = serum calcium; Ca++ = serum ionized calcium; s-P = serum phosphorus; PTH = Parathormone; iFGF23 = intact FGF23; cFGF23 = c-terminal FGF23; uCa = 24 h urinary calcium excretion; uP = 24 h urinary phosphorus excretion; Hb = Hemoglobin; TSAT = Transferrin Saturation. Bold highlights statistical significances (*p* < 0.05).

**Table 4:** Multivariate linear regression models for FGF23 isoforms with the most significant variables from our population (also reporting their 95% CI).

	**iFGF23**	
	**B (95% CI)**	***p***
s-P (mg/dL)	0.132 (0.049, 0.216)	0.002
eGFR (mL/min/m^2^)	−0.009 (−0.015, −0.003)	0.002
TNFα (pg/mL)	0.012 (0.006, 0.019)	0.003
PTH (pg/mL)	0.001 (0.000, 0.003)	0.008
	**cFGF23**	
	**B (95% CI)**	***p***
eGFR (mL/min/m^2^)	−0.010 (−0.015, −0.005)	<0.001
s-P (mg/dL)	0.117 (0.031, 0.202)	0.008
MCP-1 (pg/mL)	0.001 (0.000, 0.002)	0.038
Age (years)	0.004 (0.000, 0.009)	0.041
	**FGF23 ratio**	
	**B (95% CI)**	***p***
25-OH Vitamin D (ng/mL)	−0.005 (−0.007, −0.002)	<0.001
Age (years)	0.007 (0.003, 0.011)	<0.001
IL-6 (pg/mL)	−0.028 (−0.047, −0.010)	0.002

Notes: The three different models have considered all the parameters that previously showed statistical significance for each of intact FGF23, c-terminal FGF23, and the FGF23 ratio. VIFs were calculated to avoid collinearity in the construction of the models, and the maximum value was 2.1 for intact FGF23 with eGFR. Given that, for intact FGF23, we initially considered eGFR, PTH, s-P, 25-OH-Vit-D, TNFα, IL-6, Hb, iron, and TSAT. For c-terminal FGF23, we considered eGFR, age, s-P, transferrin, 25-OH-Vit-D, Hb, and MCP-1. For the FGF23 ratio, we considered age, PTH, 25-OH vitamin D, transferrin, TNFα, IL6, and MCP-1. Only statistically significant results are reported in this table. eGFR = estimated Glomerular Filtration Rate; s-P = serum phosphorus; PTH = Parathormone; iFGF23 = intact FGF23; cFGF23 = c-terminal FGF23; TNFα = Tumor Necrosis Factor a; MCP-1 = Monocyte Chemoattractant Protein 1; IL-6 = Interleukin 6.

## Data Availability

The dataset analyzed for this study can be found in the OSF repository accessed on 24 October 2023: https://osf.io/rchwm/?view_only=aabd1550825449feae6b748c2394e8e5.

## Supplementary Materials

The following supporting information can be downloaded at: https://www.mdpi.com/article/10.3390/jcm13133967/s1, Supplementary Figure S1: Scatter plot graphs of the correlations between eGFR and FGF23 isoforms; Supplementary Figure S2: Scatter plot graphs of the most significant correlations between FGF23 isoforms and inflammatory cytokines; Supplementary Figure S3: Scatter plot graphs of the correlations between eGFR and some inflammatory cytokines.
