# Supplementary material for: Associations of Intact and C-Terminal FGF23 with Inflammatory Markers in Older Patients Affected by Advanced Chronic Kidney Disease
Source: J Clin Med. 2024 Jul 6;13(13):3967. doi: 10.3390/jcm13133967 (PMC11242756; doi:10.3390/jcm13133967)
Supplement: Supplementary file 1 [file jcm-13-03967-s001.zip › jcm-3071240-supplementary.pdf]

Supplementary Figures

Supplementary Figure 1: Scatter plot graphs of the correlations between eGFR and FGF23 isoforms

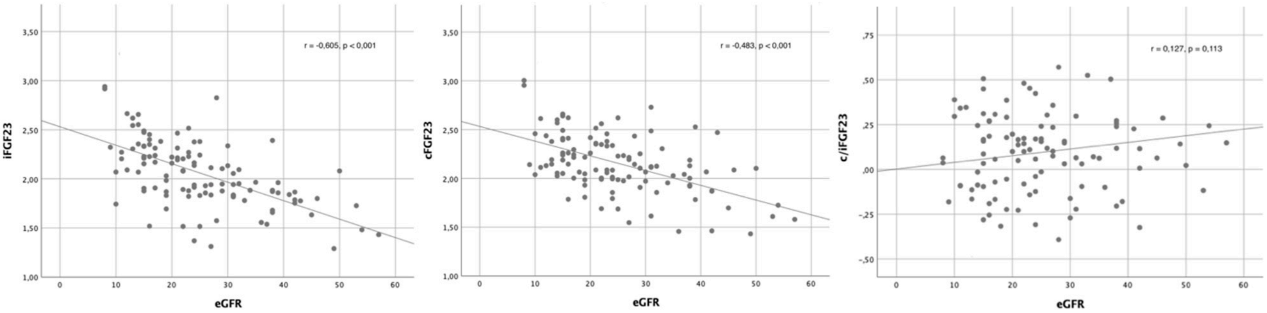

Footsteps: iFGF23; intact fibroblast growth factor 23, cFGF23; C terminal fibroblast growth factor 23, eGFR estimated glomerular filtration rate

Supplementary Figure 2: Scatter plot graphs of the most significant correlations between FGF23 isoforms and inflammatory cytokines

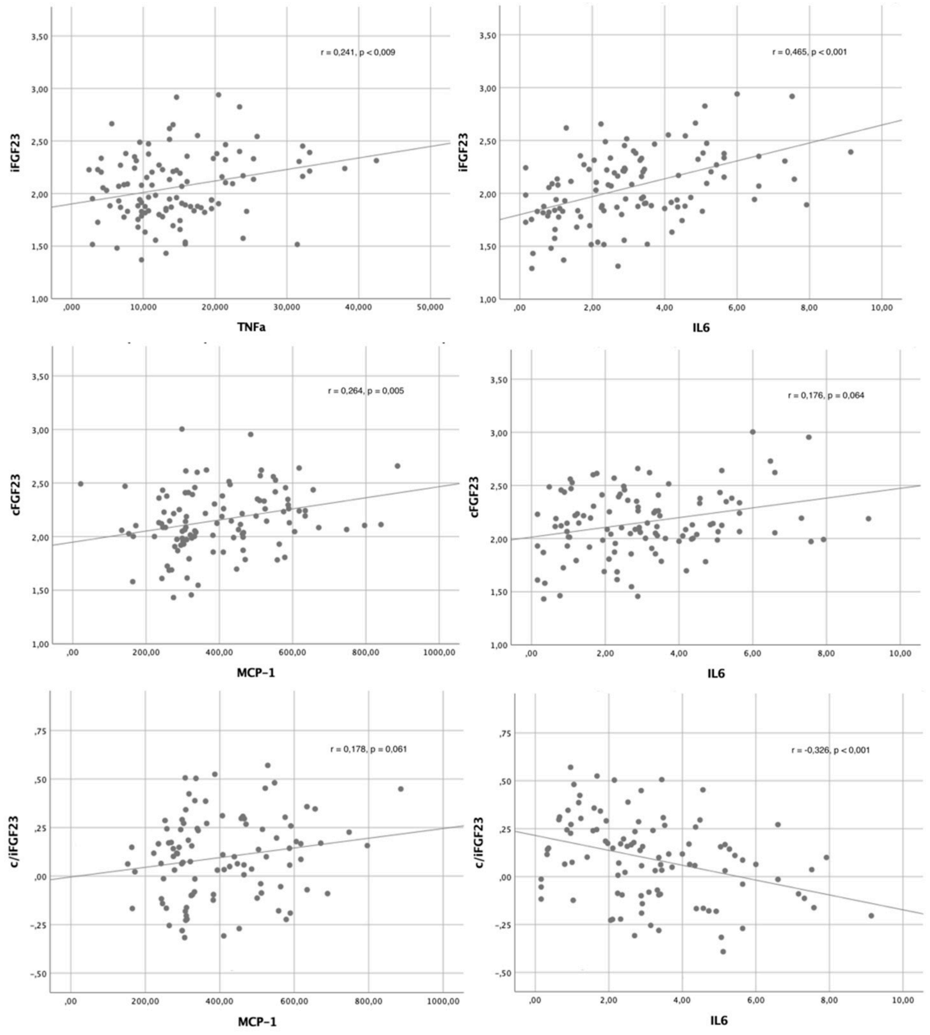

Footsteps: iFGF23; intact fibroblast growth factor 23, cFGF23; C terminal fibroblast growth factor 23, TNFα: tumor necrosis factor α; MCP-1: macrophage chemoattractant protein 1; IL-6: interleukin 6

Supplementary Figure 3: Scatter plot graphs of the correlations between eGFR and some inflammatory cytokines

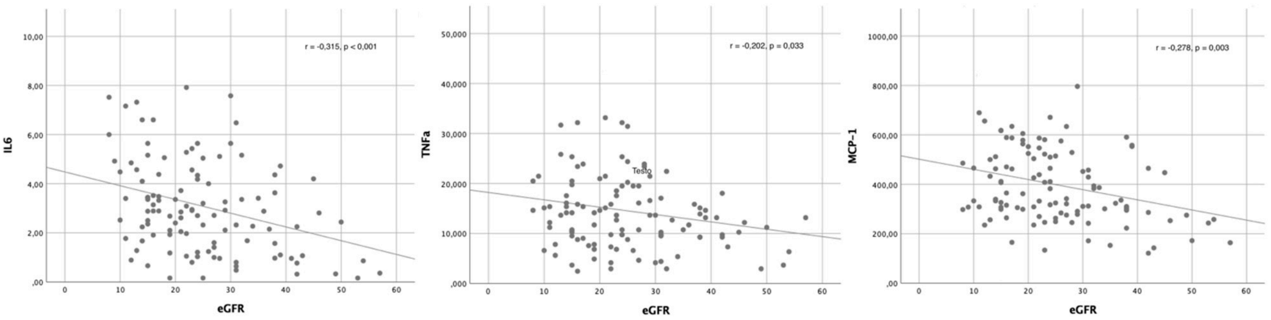

Footsteps: IL-6: interleukin 6; TNFα: tumor necrosis factor α; MCP-1: macrophage chemoattractant protein 1; eGFR: estimated glomerular filtration rate
